# Natural Language Processing and ICD-10 Coding for Detecting Bleeding Events in Discharge Summaries: Comparative Cross-Sectional Study

**DOI:** 10.2196/67837

**Published:** 2025-08-29

**Authors:** Frederic Gaspar, Mehdi Zayene, Claire Coumau, Elliott Bertrand, Marie Bettex, Marie Annick Le Pogam, Chantal Csajka

**Affiliations:** 1 Center for Research and Innovation in Clinical Pharmaceutical Sciences, Rue du Bugnon 19, Lausanne, 1011, Switzerland, 41 763306834; 2 School of Pharmaceutical Sciences, University of Geneva, Geneva, Switzerland; 3 Effixis SA, Lausanne, Switzerland; 4 Institute of Pharmaceutical Sciences of Western Switzerland, University of Geneva, Geneva and Lausanne, Switzerland; 5 Department of Epidemiology and Health Systems, Center for Primary Care and Public Health (Unisanté), University of Lausanne, Lausanne, Switzerland

**Keywords:** decision support systems, deep learning, hemorrhage, international classification of diseases, machine learning, ML, natural language processing, NLP, cross-sectional, logistic regression, bleeding, discharge summaries, adverse drug events, older adults, elderly, electronic medical records, medical records, artificial intelligence, AI, healthcare, decision-making

## Abstract

**Background:**

Bleeding adverse drug events (ADEs), particularly among older inpatients receiving antithrombotic therapy, represent a major safety concern in hospitals. These events are often underdetected by conventional rule-based systems relying on structured electronic medical record data, such as the *ICD-10 (International Statistical Classification of Diseases and Related Health Problems 10th Revision*) codes, which lack the granularity to capture nuanced clinical narratives.

**Objective:**

This study aimed to develop and evaluate a natural language processing (NLP) model to detect and categorize bleeding ADEs in discharge summaries of older adults. Specifically, the model was designed to distinguish between “clinically significant bleeding,” “severe bleeding,” “history of bleeding,” and “no bleeding,” and was compared with a rule-based algorithm using *ICD-10* codes.

**Methods:**

Clinicians manually annotated 400 discharge summaries, comprising 65,706 sentences, into four categories: “no bleeding,” “clinically significant bleeding,” “severe bleeding,” and “history of bleeding.” The dataset was divided into a training set (70%, 47,100 sentences) and a test set (30%, 18,606 sentences). Two detection approaches were developed and evaluated: (1) an NLP model using binary logistic regression and support vector machine classifiers, and (2) a traditional rule-based algorithm relying exclusively on predefined *ICD-10* codes. To address class imbalance, with most sentences categorized as irrelevant (“no bleeding”), a class-weighting strategy was applied in the NLP model. Model performance was assessed using accuracy, precision, recall, *F*_1_-score, and receiver operating characteristic (ROC) curve analyses, with manual annotations as the gold standard.

**Results:**

The NLP model significantly outperformed the rule-based approach across all evaluation metrics. At the document level, the NLP model achieved macro-average scores of 0.81 for accuracy and 0.80 for *F*_1_-score. Precision was particularly high for detecting severe (0.92) and clinically significant bleeding events (0.87), demonstrating strong classification capability despite class imbalance. ROC analyses confirmed the model’s robust diagnostic performance, yielding an area under the curve (AUC) of 0.91 when distinguishing irrelevant sentences from potential bleeding events, 0.88 for identifying historical mentions of bleeding, and notably, 0.94 for differentiating clinically significant from severe bleeding. In contrast, the rule-based *ICD-10* model demonstrated high precision (0.94) for clinically significant bleeding but poor recall (0.03) for severe bleeding events, reflecting frequent missed detections. This limitation arose due to its reliance on commonly used *ICD-10* codes (eg, gastrointestinal hemorrhage) and inadequate capture of rare severe bleeding conditions such as shock due to hemorrhage.

**Conclusions:**

This study highlights the considerable advantage of NLP over traditional *ICD-10*–based methods for detecting bleeding ADEs within electronic medical records. The NLP model effectively captured nuanced clinical narratives, including severity, negations, and historical bleeding events, demonstrating substantial promise for improving patient safety surveillance and clinical decision-making. Future research should extend validation across multiple institutions, diversify annotated datasets, and further refine temporal reasoning capabilities within NLP algorithms.

## Introduction

Adverse drug events (ADEs) are a significant patient safety issue, particularly among older adult inpatients. Globally, ADEs are estimated to affect 10%-40% of hospitalized patients, contributing to increased morbidity, mortality, and health care costs [1-3]. Among older adult patients, who are often treated using complex medication regimens, the risk of ADEs is even higher due to age-related physiological changes and a higher prevalence of polypharmacy [4,5]. Data on the incidence and impact of ADEs in Switzerland’s hospitals are sparse, however, making it difficult to fully assess the scope of the problem [6].

Antithrombotic therapy, commonly prescribed to prevent thrombotic events, significantly increases the risk of bleeding by inhibiting normal clotting mechanisms. Studies have shown that approximately 36% of older adult inpatients on antithrombotic therapy experience bleeding complications, which can lead to extended hospital stays and increased morbidity and mortality [5]. The widespread use of polypharmacy in this population further compounds the risk of drug interactions, contributing to ADEs [7]. In Swiss hospitals, the timely and accurate detection of bleeding events is considered crucial to improve patient outcomes and ensure safer care [8].

Electronic medical records (EMRs) provide an opportunity to automate the detection of ADEs such as bleeding. Bleeding events are commonly identified through structured data, particularly via the diagnostic codes in the (*ICD*) *International Classification of Diseases*, which are frequently used for billing purposes. However, *ICD* codes often lack the specificity required to capture the complexity and nuances of bleeding ADEs [9-11]. Research has shown that *ICD* codes frequently underreport ADEs, with sensitivities below 50% in many cases, leading to an incomplete picture of patient safety [12]. In addition, coding algorithms for detecting ADEs usually exhibit low sensitivity and precision, and there is no universally accepted set of *ICD-10* (*International Classification of Diseases, 10th Revision*) codes or algorithms that ensures the consistent identification of bleeding ADEs in administrative data [13]. Although a manual review of medical records can be more accurate, it is labor-intensive and impractical for widespread use [10].

Natural language processing (NLP), a branch of artificial intelligence, provides a scalable solution to the automated extraction and classification of information on bleeding ADEs from unstructured text, such as inpatient discharge summaries and clinical notes [14,15]. These notes often contain detailed narrative descriptions of clinical events, such as “nonglomerular microhematuria” or “no visible bleeding at the anamnesis,” which billing codes might miss [16]. NLP models can detect key clinical information buried within these narrative notes, providing more accurate insights into patients’ conditions than frequently used methods such as *ICD* coding. Previous studies have demonstrated that NLP can detect ADEs from clinical notes with accuracies as high as 85%-90%, significantly outperforming standard methods [17-20]. By leveraging NLP and integrating it into hospital workflows, health care professionals can improve the surveillance of ADEs, make more timely interventions, and provide more responsive, personalized patient care [21].

In this study, conducted within the framework of the Swiss Monitoring of Adverse Drug Events (SwissMADE) project [8], we hypothesized that an NLP-based approach would be more effective than *ICD* code–based algorithms for detecting and categorizing bleeding ADEs among older adult inpatients receiving antithrombotic therapy at Lausanne University Hospital. The primary objective was to develop an NLP model capable of identifying bleeding ADEs from the discharge summaries of older adult inpatients hospitalized in 2015 and 2016 and to categorize these events based on their timing (ie, before admission or during the hospital stay) and severity (clinically significant bleeding or severe bleeding). The secondary objective was to compare the NLP model’s performance against standard *ICD-10*–based algorithms and identify the most effective automated method for detecting bleeding ADEs in Switzerland’s health care context.

## Methods

### Study Design

**Figure 1.**
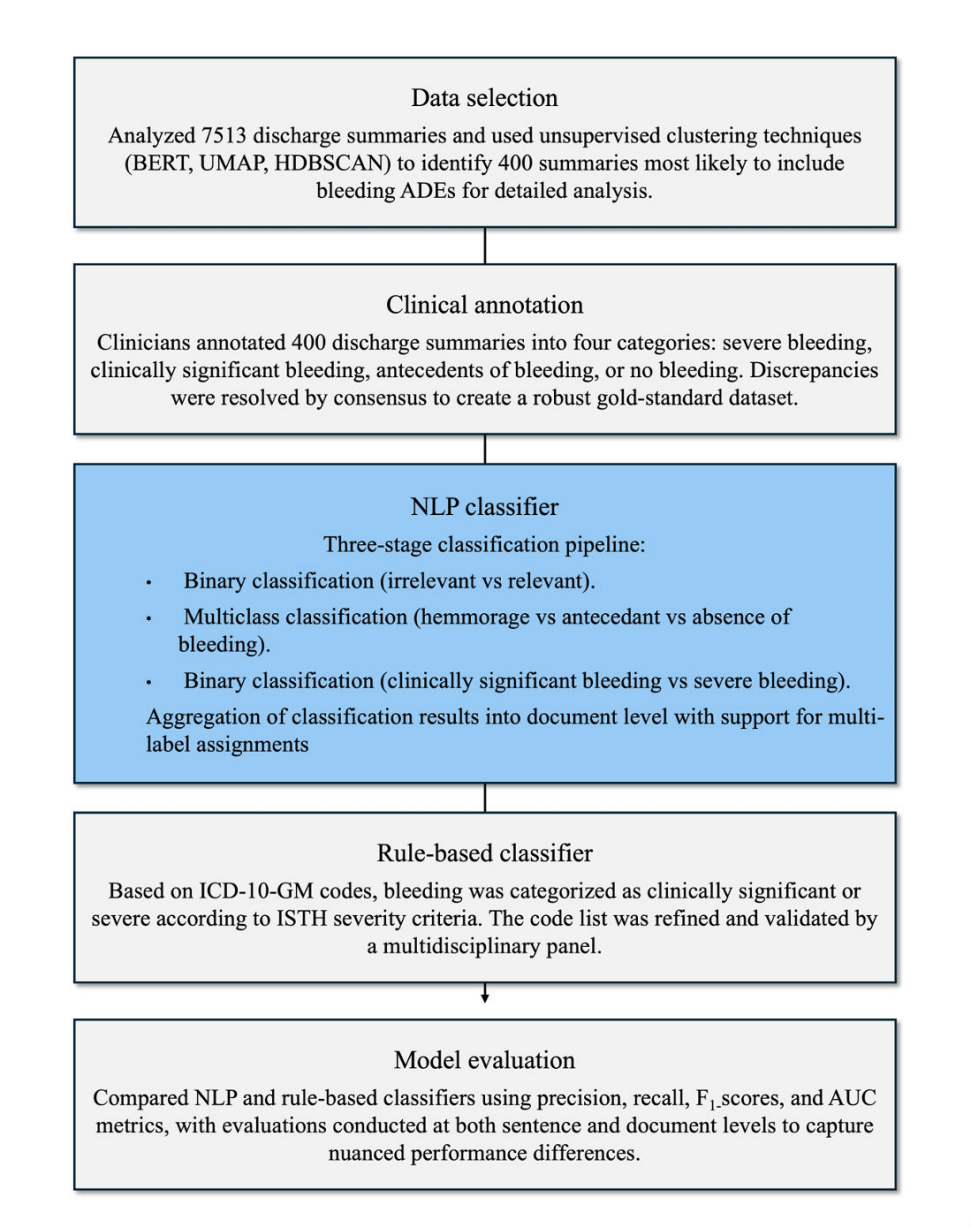
Overview of the methodological framework used in this study. ADE: adverse drug event; AUC: area under the curve; BERT: Bidirectional Encoder Representations from Transformers; HDBSCAN: hierarchical density-based spatial clustering of applications with noise; *ICD-10-GM*: *German Modification of the International Statistical Classification of Diseases and Related Health Problems, 10th Revision*; ISTH: International Society on Thrombosis and Haemostasis; NLP: natural language processing; UMAP: uniform manifold approximation and projection.

We conducted a secondary analysis of unstructured data in the EMRs investigated by the SwissMADE study, a multicenter, cross-sectional study that used retrospective medical data from 4 large Swiss hospitals [8]. Figure 1 provides an overview of the methodological framework used in this study.

### Study Population and Dataset Selection

The dataset comprised the discharge summaries of patients aged 65 or older who were hospitalized for more than 24 hours in 2015 and 2016 and received at least one antithrombotic medication during their stay. These summaries also included administrative data, such as *ICD-10-GM* (*International Classification of Diseases, 10th Revision, German Modification*) diagnostic codes. A detailed description of the SwissMADE study’s methods has been published previously [8].

Of the 7513 discharge summaries examined, an unsupervised machine learning approach identified 400 as likely to contain bleeding ADEs (Figure S1 in Multimedia Appendix 1 ). This approach involved text scanning, thematic aggregation, and data extraction. The study generated unique sentence embeddings by integrating Bidirectional Encoder Representations from Transformers (BERT) into the Sentence Transformer library [22]. Techniques such as Uniform Manifold Approximation and Projection (UMAP) [23] and Hierarchical Density-Based Spatial Clustering of Applications with Noise (HDBSCAN) [24] were applied to organize these embeddings into “clusters” of bleeding ADEs. This methodology was instrumental in selecting the 400 discharge summaries most relevant to the study.

### Annotation of Clinical Documents

The 400 discharge summaries were first annotated by clinicians and then divided into a training set (n=280) and a test set (n=120). The distribution of summaries was randomized to ensure the sample remained representative of the overall population of hospitalized patients.

Three clinicians independently annotated each discharge summary using four predefined labels:

1. Presence of severe bleeding: this label was used when a discharge summary explicitly identified severe bleeding, either by using the term “severe” or by describing conditions that meet the criteria for severe bleeding, such as fatal bleeding, bleeding at critical sites (eg, intracranial and intraspinal), a drop in hemoglobin of ≥20 g/L, or transfusion of ≥2 units of blood, as defined by the International Society on Thrombosis and Haemostasis (ISTH) [25].
2. Presence of clinically significant bleeding: this label was used when bleeding was mentioned in the clinical documentation but did not meet the criteria for severe bleeding.
3. History of bleeding: this label was applied when a discharge summary mentioned bleeding in the patient’s medical history before their hospital admission.
4. Absence of bleeding: this label was used when a discharge summary did not mention bleeding.

A fourth clinician resolved any disagreements, and this classification was used as the gold standard for training the machine learning model. Fleiss kappa coefficient, calculated from 30 summaries, showed 96% agreement among clinicians, allowing a shift to a single-reviewer approach. Only discharge summaries signed by an attending physician were included to ensure data credibility.

### Development of the NLP-Based Classifier

The development method comprised three phases: segmenting discharge summaries into sentences, classifying those sentences, and aggregating them at the document level.

#### Phase 1: Segmentation

Sentences were segmented from the discharge summaries using the pretrained French spaCy model (Explosion AI) [19], chosen for its efficiency, robustness, and widespread adoption in NLP pipelines [26]. Given that sentence segmentation is a standard preprocessing step with minimal differences among comparable models [27], no additional comparative analyses were performed. To reduce noise, sentences with fewer than three characters were excluded, a decision supported by pilot tests demonstrating minimal loss of meaningful content.

#### Phase 2: Classification Process

The classification process addressed the challenge of class imbalance, particularly at the sentence level, where the majority of sentences were labeled as “Irrelevant,” indicating no bleeding-related information.

To mitigate this imbalance during training, a class-weighting strategy was applied in the logistic regression model used for the initial binary classification [28]. This approach adjusted the contribution of each class to the loss function by assigning higher weights to minority classes, such as “severe bleeding,” and lower weights to the majority class, “Irrelevant.” This adjustment improved the model’s ability to identify rare but clinically critical cases. Additional details on the dataset preparation and the class-weighting strategy are provided in the supplementary materials.

**Figure 2.**
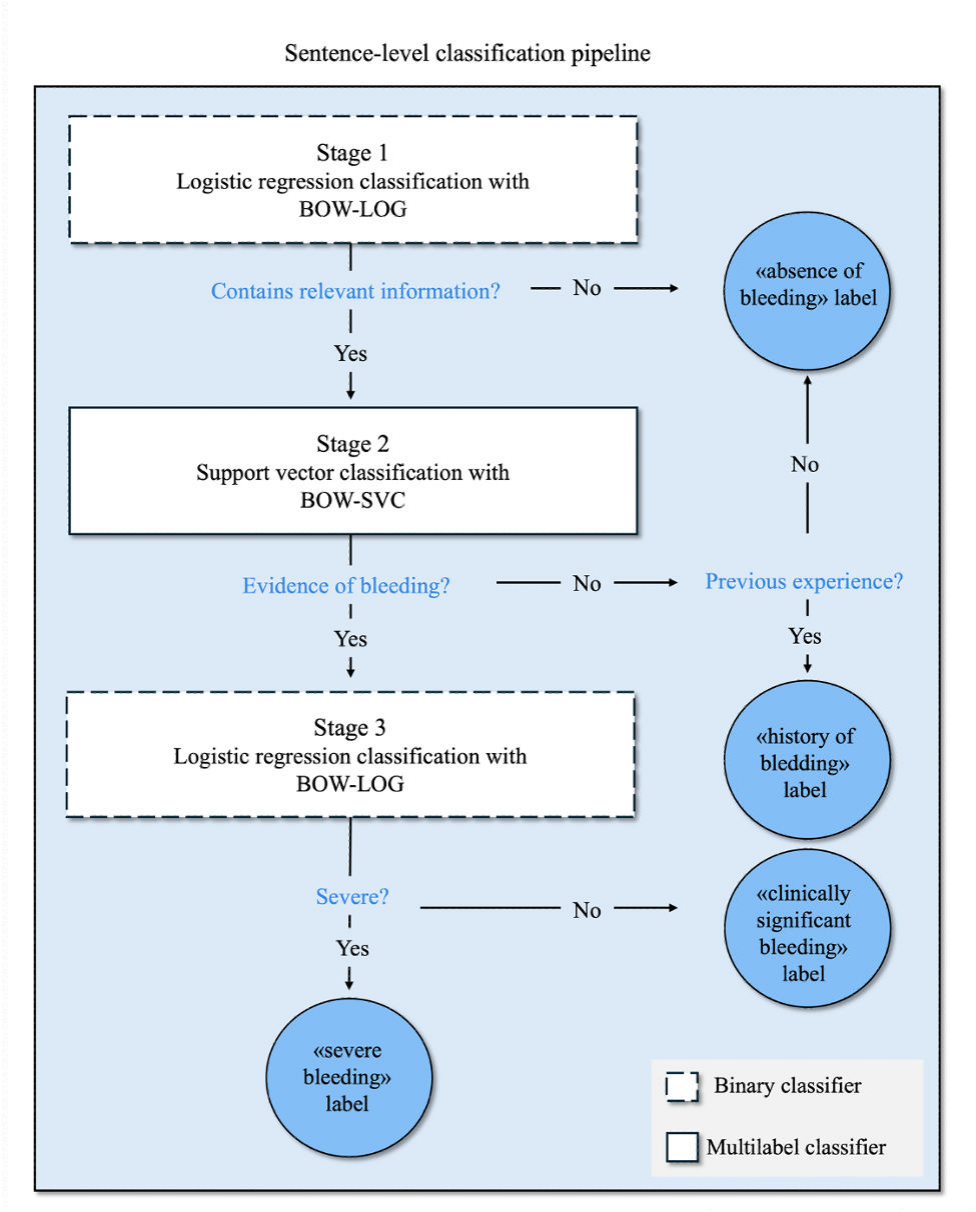
Sentence-level classification pipeline for detecting bleeding events. In this pipeline: BOW-LOG: logistic regression classification using a bag-of-words (BOW) model to determine whether the sentence contains bleeding-relevant information; BOW-SVM: support vector machine (SVM) classification using a BOW model to assess whether there is evidence of bleeding in the sentence; BOW-LOG: a second logistic regression classification using a BOW model to evaluate the severity of the bleeding event (ie, whether it is severe or clinically significant).

Figure 2 illustrates the multistage classification process for identifying bleeding ADEs in clinical narratives. Stage 1 used a binary logistic regression model to classify sentences as either containing bleeding-related information (labeled “relevant;” value=1) or not (“irrelevant;” value=0), reducing the number of nonrelevant sentences. Stage 2 used a support vector machine (SVM) classifier to further divide the relevant sentences into three categories: “irrelevant,” “antecedent,” or “bleeding-related.” Stage 3 applied a second binary classification to “bleeding-related” sentences, categorizing them as either “clinically significant” (value=0) or “severe” (value= 1).

Logistic regression and a bag-of-words–based SVM were selected due to their simplicity, interpretability, and effectiveness on smaller datasets, minimizing the risk of overfitting compared to more complex deep learning methods. Preliminary experiments using deep learning models yielded poor performance, likely due to the limited size of the dataset; therefore, these methods were not pursued further in this study.

The entire process used bag-of-words encoding to convert the text into a format suitable for machine learning algorithms. Robustness was ensured through 5-fold cross-validation [29], regularization techniques (eg, L2 regularization for logistic regression and optimization of the penalty parameter [C] in SVM), and hyperparameter tuning via grid search [30,31]. These methodological choices optimized performance while preventing overfitting.

#### Phase 3: Document Aggregation

Sentence-level classification results were aggregated at the discharge summary level by grouping sentences under their corresponding document ID and combining the predictions using a union-like operation. If all sentences in a document were labeled “irrelevant,” the entire document was classified the same. Otherwise, the document was assigned one or more of the following labels: “antecedent,” “clinically significant bleeding,” or “severe bleeding.” Unlike sentences, documents could receive multiple labels.

### Rule-Based Classifier Development

In parallel with the NLP approach, we developed a rule-based classifier using *ICD-10-GM* codes to detect bleeding ADEs. This classifier enabled us to compare the analysis with the NLP methods. We began by compiling a comprehensive list of *ICD-10* diagnostic codes related to bleeding, drawing on subdivisions defined by the ISTH and codes identified in previous studies [32-34].

We thoroughly explored *ICD-10* ontologies to identify additional codes for terms such as “bleeding” and “hemorrhage.” A multidisciplinary panel of physicians, pharmacologists, pharmacists, and statisticians reviewed and expanded this list, adding codes for conditions such as hemodynamic instability, drug-induced bleeding, and contusions. We then categorized these codes into two mutually exclusive groups based on the ISTH’s severity criteria: “clinically significant bleeding” and “severe bleeding.” The classification considered factors such as the site of bleeding.

The complete list of codes used appears in Tables S1 and S2 in Multimedia Appendix 1. However, unlike the NLP approach, the rule-based method was limited to just 2 labels due to the absence of specific *ICD-10* codes for identifying a patient’s history of bleeding. Moreover, the rule-based approach did not account for timing, making it difficult to determine whether bleeding occurred before or during the hospital stay.

### Model Evaluation and Comparison

We conducted a comparative analysis of the rule-based classifier and the NLP method to assess their effectiveness and accuracy in identifying hemorrhagic events from discharge summaries. Using the independent test dataset to ensure unbiased assessments, we applied standardized evaluation metrics, namely precision, recall, specificity, and *F*_1_-score, focusing on each method’s ability to detect clinically significant and severe bleeding. We used a receiver operating characteristic (ROC) curve to evaluate the NLP model’s diagnostic capacity, measuring its overall performance through its area under the curve (AUC) [35]. We also calculated Cohen kappa to facilitate a comparative analysis of the methods’ detection accuracies [36].

All statistical analyses were performed using Python software (version 3.9, Python Software Foundation), ensuring a robust computational environment. We calculated both micro- and macro-averages to provide a comprehensive evaluation of the classifiers’ performances. Micro-averages were computed at the sentence level, measuring overall performance across all sentences, while macro-averages were calculated at the document level, giving equal weight to each document regardless of the number of sentences it contained.

### Ethical Considerations

This study involved secondary analysis of pre-existing clinical data from discharge summaries collected within the SwissMADE project, a multicenter, retrospective, cross-sectional study approved by the Swiss Ethics Committees (CER-VD, No. 2016‐02008). This analysis used retrospective data from EMRs collected within the SwissMADE study. The original study protocol, approved by the ethics committee, included a waiver of informed consent. Due to the narrative nature of the clinical data, complete anonymization was not feasible. Strict confidentiality measures were implemented, including restricted data access, secure data handling, and reporting of results in aggregate form only, in compliance with Swiss federal and institutional data protection standards. No patient compensation was provided, as the study involved secondary analysis of pre-existing clinical data. No identifiable images or other materials from individual patients were included in this paper or its supplementary materials. Consequently, there was no requirement for obtaining consent from individuals for use of identifiable images.

## Results

### Overview

A total of 400 discharge summaries were analyzed, comprising 65,706 annotated sentences. Of these, 47,100 sentences were allocated to the training set and 18,606 to the test set. Detailed demographic and clinical characteristics of the hospital stays associated with each dataset are presented in Table 1.

**Figure 3.**
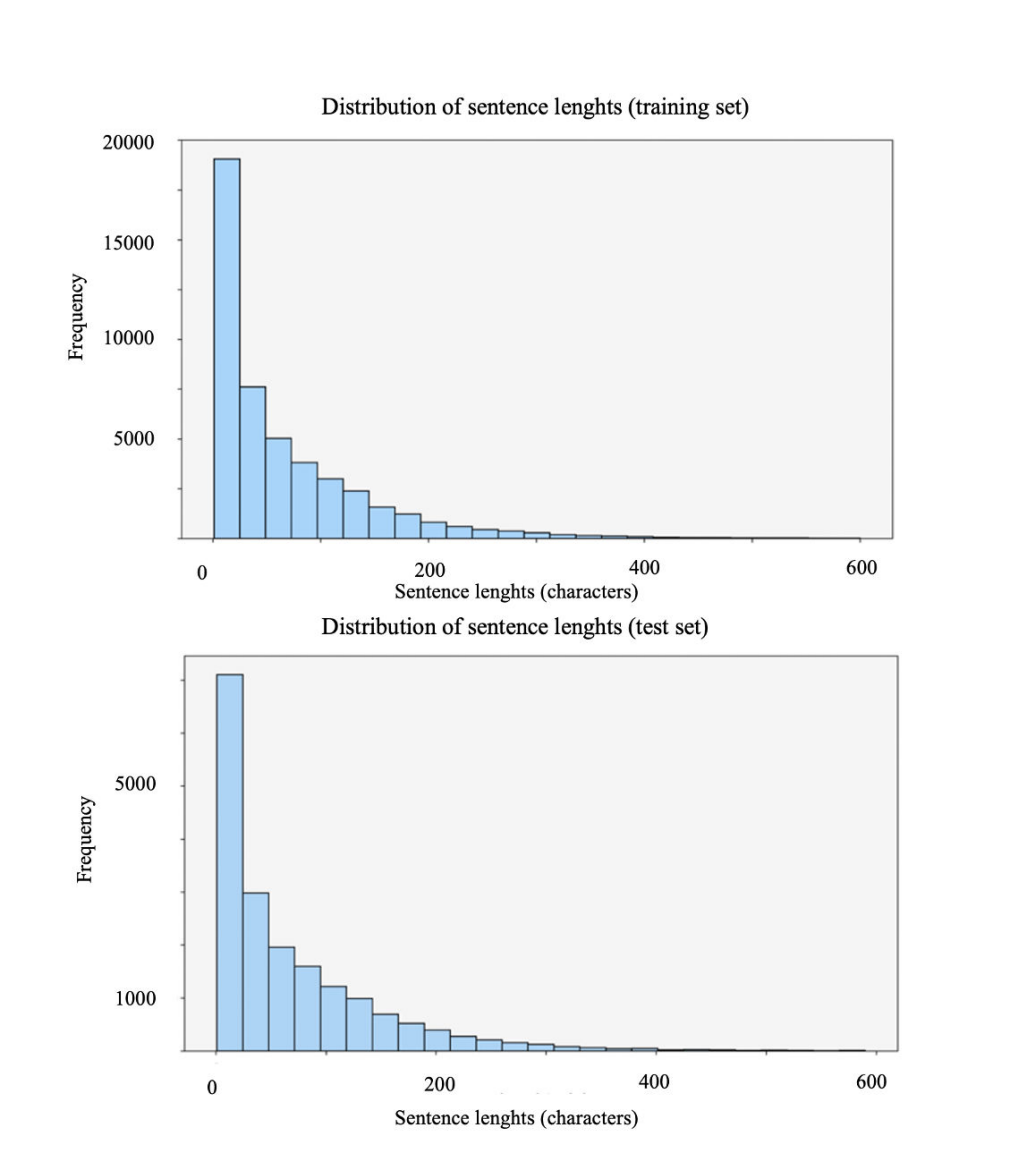
Distribution of sentence lengths in training and test sets.

The distribution of sentence lengths in Figure 3 reveals a right-skewed pattern, with most sentences under 100 characters. To reduce noise, sentences shorter than 3 characters were excluded, accounting for 2.72% of the dataset, ensuring more meaningful content for robust model training and evaluation.

Sentence-level analysis revealed a predominance of “irrelevant” annotations, reflecting the large amount of information in discharge summaries unrelated to bleeding. However, class distribution was more balanced at the document level, demonstrating the complexity of clinical documentation, where multiple annotations often coexist within a single summary. Table 2 provides the detailed distribution of these categories.

The NLP model demonstrated strong classification capabilities, achieving over 85% accuracy across all categories at the document level. It also showed robust performance, with a precision exceeding 72% across categories and a recall of 98% for “irrelevant” instances. *F*_1_-scores indicated balanced performance despite class imbalances, highlighting the model’s ability to manage diverse data distributions. A detailed summary of the performance metrics for our multilabel classification model is provided in Table 3.

**Figure 4.**
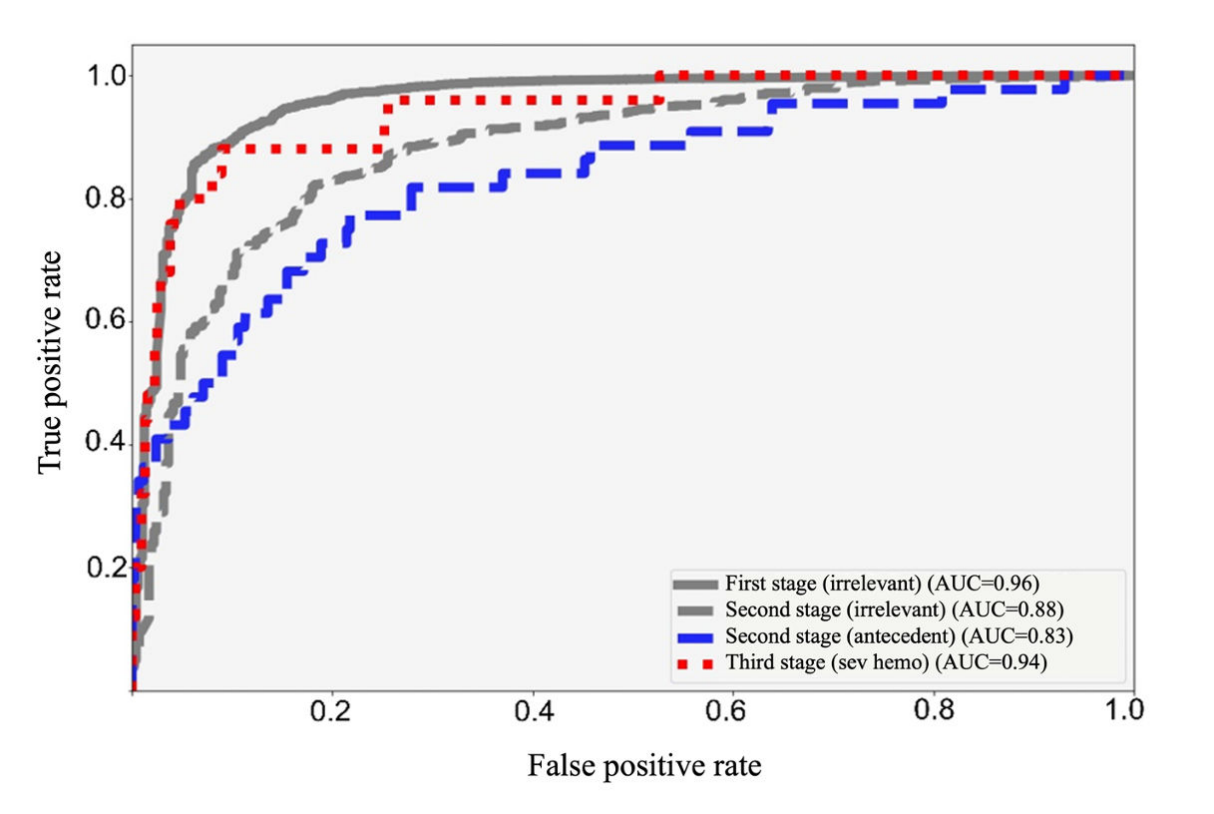
Receiver operating characteristic (ROC) curves showing the diagnostic performance of the multistage classification model at various thresholds, illustrating the trade-off between the true positive rate (sensitivity) and the false positive rate (1 - specificity).

As shown in Figure 4, ROC curve analysis further highlighted the model’s diagnostic accuracy. In stage one, the model achieved an AUC of 0.91 for classifying sentences as either “irrelevant” or “potentially bleeding-related,” effectively filtering out irrelevant data. In stage 2, it refined these classifications into the “irrelevant” and “antecedent” categories, with AUCs of 0.88 and 0.83, respectively. Stage 3 focused on distinguishing “clinically significant bleeding” from “severe bleeding,” achieving an AUC of 0.94. Overall, the ROC curves demonstrated the model’s consistently high performance across all stages, with elevated AUC values reflecting its strong ability to distinguish between classes.

The rule-based classifier, while simpler than the multilabel NLP model, showed high precision in identifying clinically significant bleeding events. Table 4 provides a detailed comparison of the algorithm’s metrics and those of the NLP model.

**Table 1.** Training and testing set patients’ characteristics.

Variable	Training set	Test set
Discharge summaries, n (%)	280 (70)	120 (30)
Sentences, n (%)	47100 (72)	18606 (28)
Unique patients, n (%)	270 (96)	120 (100)
Length of stay (days), median (IQR)	15 (8–28)	13 (7-22)
Female, n (%)	111 (40)	89 (74)
Age (years), median (IQR)	81 (74-87)	79 (72-85)
ICU^a^ admissions, n (%)	17 (6)	4 (3)
Modes of admission, n (%)		
Emergency	214 (76)	89 (74)
Planned,	49 (18)	20 (17)
Internal transfer	15 (5)	6 (5)
Transfer within 24 hours	2 (1)	5 (4)

a ICU: intensive care unit.

**Table 2.** Class distribution in the training and test sets at the sentence and document levels.

Classification label	Sentence level^a^		Document level^b^	
	Training set, n (%)	Test set, n (%)	Training set, n (%)	Test set, n (%)
Irrelevant (absence of bleeding)	45897 (97.45)	18118 (97.38)	103 (36.79)	44 (36.67)
History of bleeding	154 (0.33)	58 (0.31)	67 (23.93)	22 (18.33)
Clinically significant bleeding	900 (1.91)	373 (2.00)	141 (50.36)	60 (50.00)
Severe bleeding	149 (0.32)	57 (0.31)	77 (27.50)	31 (25.83)

a Sentence level: frequency and proportion of each classification label per individual sentence.

b Document level: frequency and proportion of documents containing at least one instance of the respective classification label.

**Table 3.** Detailed performance metrics of the multilabel classification model.

Metric	Irrelevant	History of bleeding	Clinically significant bleeding	Severe bleeding	Macro-average^a^	Micro-average^b^
Accuracy	0.83	0.68	0.86	0.89	0.81	0.84
Precision	0.81	0.72	0.87	0.92	0.83	0.85
Recall	0.98	0.88	0.59	0.31	0.69	0.71
*F*_1_-score	0.89	0.65	0.88	0.70	0.78	0.80

a Macro-average: average performance across document-level classifications.

b Micro-average: average performance across sentence-level classifications.

**Table 4.** Detailed performance metrics of the rule-based classification model.

Metric	Irrelevant	Antecedent^a^	Clinically significant bleeding	Severe bleeding	Macro-average	Micro-average^b^
Accuracy	0.81	—^c^	0.86	0.74	0.80	—
Precision	0.80	—	0.94	0.50	0.75	—
Recall	0.95	—	0.77	0.03	0.58	—
*F*_1_-score	0.87	—	0.84	0.06	0.59	—

a Metrics for the “Antecedent” category are not provided due to the absence of corresponding *International Statistical Classification of Diseases and Related Health Problems, 10th Revision* codes.

b “Micro-average” was not calculated as the rule-based model uses *International Statistical Classification of Diseases and Related Health Problems, 10th Revision* codes linked to hospital stays.

c Not available.

The rule-based classifier achieved a precision score of 0.94 for “clinically significant bleeding,” highlighting its accuracy in detecting these events. However, its performance in identifying “severe bleeding” was significantly weaker, with a recall of only 0.03. This low recall indicates that while the model could detect severe bleeding when present, it also frequently missed such events.

**Figure 5.**
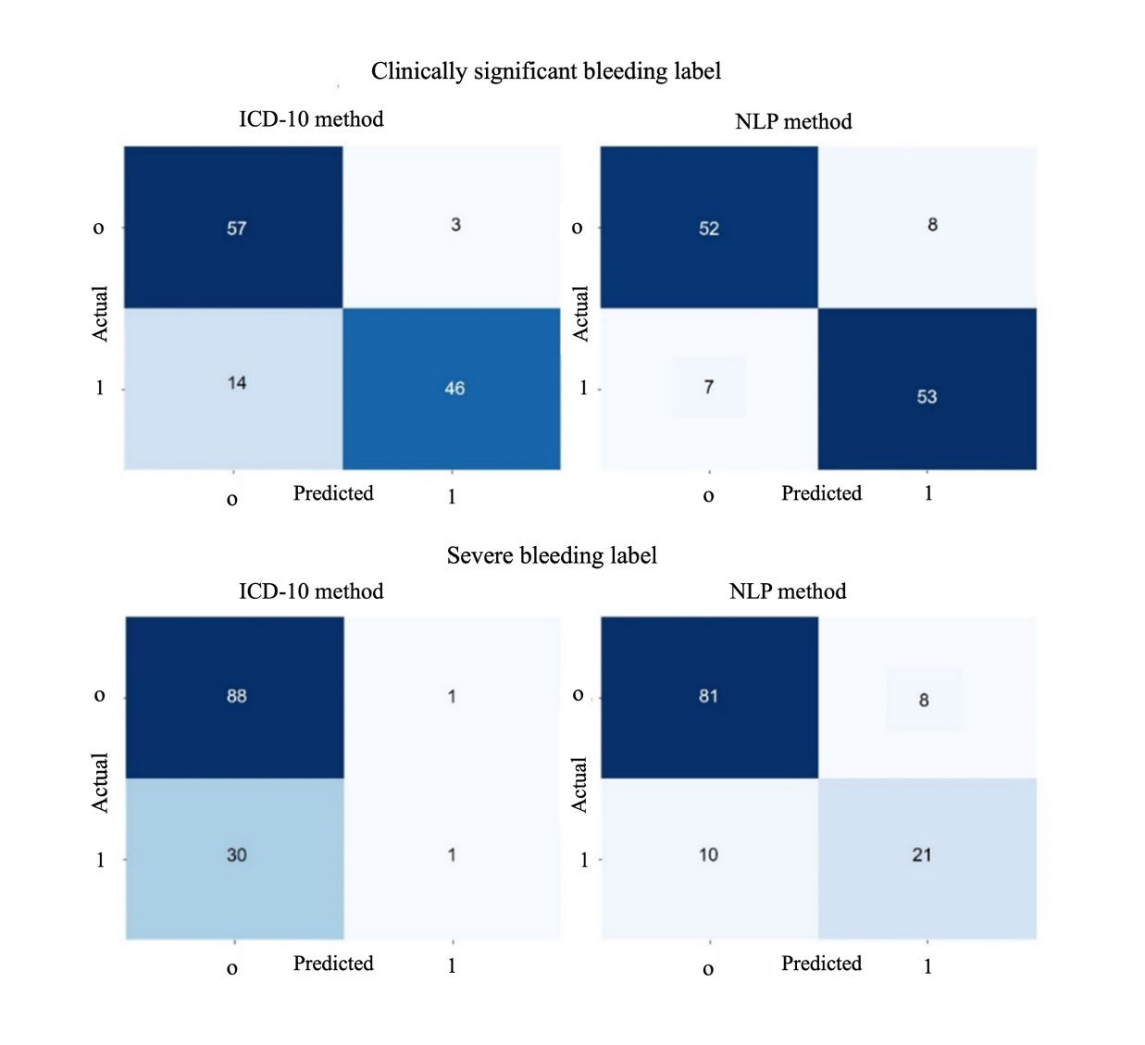
Comparative analysis of confusion matrices for bleeding detection using natural language processing (NLP) and *ICD* (*International Classification of Diseases*) coding methods. This figure contrasts the performance of the NLP model and the *ICD* coding approach, displaying the counts of true positives, true negatives, false positives, and false negatives, thus providing a comparative evaluation of both methods. *ICD-10*: *International Statistical Classification of Diseases and Related Health Problems, 10th Revision*.

For “clinically significant bleeding,” the classifier relied heavily on frequently used *ICD-10* codes, such as K92.2 (gastrointestinal hemorrhage, unspecified), R31 (hematuria, unspecified), and K26.4 (gastric ulcer, acute with hemorrhage), which contributed to its high precision. In contrast, codes associated with “severe bleeding,” including R57.1 (shock due to hemorrhage) and I85.3 (esophageal varices with bleeding), were less common in the dataset, resulting in poorer performance for this category. The rule-based model achieved an *F*_1_-score of 0.84 for “clinically significant bleeding” but only 0.06 for “severe bleeding,” underscoring the disparity in its ability to handle these 2 categories. Figure 5 highlights the comparative performance of the NLP and rule-based models in detecting clinically significant and severe bleeding. The NLP model consistently demonstrated higher recall and balanced classification across both categories, effectively addressing limitations observed in the rule-based approach.

## Discussion

### Principal Findings

This study developed and validated an NLP-based model for detecting bleeding events from clinical narratives, achieving a high level of accuracy. The model demonstrated 91% sentence-level accuracy and 88% document-level accuracy. Compared to traditional *ICD-10* code–based methods, the NLP model provided more nuanced and precise detection of bleeding events, effectively capturing details that *ICD-10* codes often miss, particularly in cases involving secondary conditions or multiple types of bleeding ADEs. These findings underscored NLP’s potential to improve the detection and management of adverse events.

One of the model’s primary strengths lies in its high precision and recall, even in the presence of class imbalance, particularly when differentiating between “clinically significant” and “severe bleeding.” It performed well in interpreting complex clinical data, including negations (eg, “no evidence of bleeding”) and secondary conditions, which are typically challenging for rule-based *ICD-10* approaches [9]. By integrating these nuances, the NLP model provided a more comprehensive understanding of patients’ clinical status. Its ability to analyze unstructured clinical narratives highlights its potential for real-time decision-making, enabling clinicians to identify bleeding events more accurately and promptly, ultimately improving patient outcomes in hospital settings [10,11,37].

### Comparison With Prior Work

The model’s accuracy, precision, recall, and *F*_1_-scores compare favorably with those reported in the literature, including models based on deep learning architectures such as biLSTM-CRF or transformers such as BERT [38]. However, most of these studies use larger English-language datasets and cover a broader range of clinical events. Differences in language (French vs English), dataset size, annotation schemes, and outcome definitions make direct comparisons difficult. While transformer-based models have shown excellent performance in clinical NLP [39,40], their computational demands and limited interpretability may hinder real-world implementation [41]. In this study, we prioritized interpretable and efficient models, such as logistic regression and SVM, offering a favorable trade-off between performance and usability. Future efforts may explore hybrid frameworks that combine advanced performance with interpretability [42].

A distinctive advantage of our approach is the use of multilabel classification, which allows the model to detect co-occurring conditions, such as clinically significant and severe bleeding, or current and historical bleeding, within a single document. This contrasts with most previous studies that rely on single-label classifiers and enhance adaptability to real-world clinical scenarios. However, the model still faced challenges with temporal reasoning, particularly in distinguishing recent events from past ones, underscoring the need for more advanced temporal analysis techniques [43-45].

The limitations of *ICD-10* coding, particularly its inability to reflect clinical nuances, are well documented in the literature. As previously observed by Johnson et al [46], reliance on a small number of broad codes, such as K92.2 (gastrointestinal bleeding, unspecified) and R57.1 (shock due to bleeding), likely contributed to low recall for severe bleeding. *ICD-10* lacks the granularity to differentiate historical versus active bleeding, mild versus severe presentations, or to correctly interpret negations. Furthermore, *ICD* codes are primarily designed for billing and administrative purposes, contributing to underreporting or misclassification of bleeding ADEs [9-11,17]. These limitations further support the relevance of NLP approaches, which offer greater flexibility and contextual understanding.

Negation handling was a particular strength of our model. Where many previous approaches have struggled, our model correctly interpreted expressions such as “no source of bleeding” [47], substantially reducing false positives and enhancing clinical utility [48]. In hospital settings, accurate interpretation of negation is essential to avoid unnecessary investigations or treatments [49].

### Limitations

This study had several limitations that should be acknowledged. First, the dataset consisted solely of discharge summaries from Lausanne University Hospital, which may limit its generalizability. Data from a single large tertiary care institution might not represent the variety of clinical settings and regions in which the model could be deployed. In addition, the over-representation of certain *ICD-10* codes, such as K92.2, likely contributed to the model’s high precision for detecting clinically significant bleeding. Expanding the baseline dataset could help improve the model’s robustness and ability to generalize across different hospital environments [50]. Future work should therefore focus on expanding the dataset with discharge summaries from multiple hospitals, enabling broader validation and assessment of the model’s temporal robustness and applicability in diverse health care environments.

Second, class imbalance in the dataset, particularly the limited number of “severe bleeding” cases, posed a challenge. While the model performed well overall, its detection of rare events was enhanced by applying class weighting during training, adjusting the contribution of each class to the loss function. This strategy improved detection of underrepresented but clinically important categories without compromising overall performance. Further improvements could involve oversampling techniques, synthetic data generation, or advanced loss functions such as the segmented harmonic loss [51,52]. Domain-specific keyword-enhanced classification may also refine the model’s ability to identify severe bleeding [53]. Despite these possible improvements, the current class-weighting strategy and multistage framework offered a robust and interpretable solution suited for deployment in resource-constrained health care settings.

Third, despite satisfactory overall performance, the model’s accuracy for detecting severe bleeding dropped to around 70%. This decrease was largely due to the model’s tendency to overinterpret numerical data (eg, hemoglobin and hematocrit values) as indicative of severe bleeding, particularly when such values appeared near bleeding-related terms. These misclassifications led to false positives and suggest a need for improved contextual differentiation between clinically relevant data and incidental numeric values. The model also struggled to capture the timing of bleeding events, a critical limitation in clinical decision-making, where understanding whether a condition is active or historical can influence diagnosis and treatment [54]. Future research should aim to enhance contextual differentiation and temporal reasoning capacities within NLP models.

Finally, the study was restricted to data from 2015 to 2016 due to the reliance on high-quality, manually annotated data from the SwissMADE project, making our analysis primarily a proof-of-concept. Future studies should integrate more recent clinical discharge summaries to validate temporal robustness further and ensure the model remains applicable in evolving health care environments [55].

### Future Directions

NLP models, particularly those using deep learning or transformer-based architectures, require significant computational resources for training and deployment. Although our model was relatively efficient, scalability remains challenging, particularly for real-time clinical applications requiring continuous updates and large datasets. Furthermore, although integrating large language models such as GPT-3 (OpenAI) or BERT holds the promise of improved performance, it also introduces concerns around computational cost and the secure handling of sensitive patient data [50,56-59]. These practical challenges will have to be addressed before the widespread adoption of NLP models in clinical settings [54,60].

### Conclusions

Despite some limitations, this study adds to the growing evidence supporting the use of NLP for detecting ADEs such as bleeding. The model outperformed standard *ICD-10*–based approaches by capturing nuanced clinical information often missed in structured data, including negations and secondary conditions. The use of multilabel classification improved its flexibility, allowing it to handle overlapping bleeding events in complex clinical scenarios. These features position NLP as a promising tool for enhancing real-time clinical decision-making and patient safety. Future work should focus on expanding the dataset to include records from multiple hospitals and care settings, improving generalizability. Integrating additional data sources, such as laboratory results, imaging, and progress notes, and exploring advanced NLP techniques such as BERT or GPT could further improve accuracy and temporal reasoning. Validating the model across diverse clinical environments and combining structured with unstructured data will be essential to build robust tools for bleeding ADE detection and support broader clinical implementation.

## Supplementary material

10.2196/67837 Multimedia Appendix 1 A comparative cross-sectional study of natural language processing and *ICD-10* (*International Classification of Diseases, 10th Revision*) coding for detecting bleeding events in discharge summaries.

## Abbreviations

ADE: adverse drug event
AUC: area under the curve
BERT: Bidirectional Encoder Representations from Transformers
EMR: electronic medical record
HDBSCAN: Hierarchical Density-Based Spatial Clustering of Applications with Noise
*ICD*: *International Classification of Diseases*
*ICD-10*: *International Statistical Classification of Diseases and Related Health Problems 10th Revision*
*ICD-10-GM*: *German Modification of the International Statistical Classification of Diseases and Related Health Problems, 10th Revision*
ISTH: International Society on Thrombosis and Haemostasis
NLP: natural language processing
ROC: receiver operating characteristic
SVM: support vector machine
SwissMADE: Swiss Monitoring of Adverse Drug Events
UMAP: Uniform Manifold Approximation and Projection

## References

[1] Cook DJ, Griffith LE, Walter SD (2001). The attributable mortality and length of intensive care unit stay of clinically important gastrointestinal bleeding in critically ill patients. Crit Care.

[2] Krähenbühl-Melcher A, Schlienger R, Lampert M, Haschke M, Drewe J, Krähenbühl S (2007). Drug-related problems in hospitals: a review of the recent literature. Drug Saf.

[3] Berger JS, Bhatt DL, Steg PG (2011). Bleeding, mortality, and antiplatelet therapy: results from the Clopidogrel for High Atherothrombotic Risk and Ischemic Stabilization, Management, and Avoidance (CHARISMA) trial. Am Heart J.

[4] Classen DC, Pestotnik SL, Evans RS, Burke JP (1991). Computerized surveillance of adverse drug events in hospital patients. JAMA.

[5] Kanagaratnam L, Abou Taam M, Heng M, De Boissieu P, Roux MP, Trenque T (2015). Serious adverse drug reaction and their preventability in the elderly over 65 years. Therapie.

[6] Beeler PE, Stammschulte T, Dressel H (2023). Hospitalisations related to adverse drug reactions in Switzerland in 2012-2019: characteristics, in-hospital mortality, and spontaneous reporting rate. Drug Saf.

[7] Long SJ, Brown KF, Ames D, Vincent C (2013). What is known about adverse events in older medical hospital inpatients? A systematic review of the literature. Int J Qual Health Care.

[8] Gaspar F, Lutters M, Beeler PE (2022). Automatic detection of adverse drug events in geriatric care: study proposal. JMIR Res Protoc.

[9] Wilchesky M, Tamblyn RM, Huang A (2004). Validation of diagnostic codes within medical services claims. J Clin Epidemiol.

[10] Bates DW, Evans RS, Murff H, Stetson PD, Pizziferri L, Hripcsak G (2003). Detecting adverse events using information technology. J Am Med Inform Assoc.

[11] Hohl CM, Karpov A, Reddekopp L, Doyle-Waters M, Stausberg J (2014). ICD-10 codes used to identify adverse drug events in administrative data: a systematic review. J Am Med Inform Assoc.

[12] Hazlehurst B, Mullooly J, Naleway A, Crane B (2005). Detecting possible vaccination reactions in clinical notes. AMIA Annu Symp Proc.

[13] Shehab N, Ziemba R, Campbell KN (2019). Assessment of ICD-10-CM code assignment validity for case finding of outpatient anticoagulant-related bleeding among Medicare beneficiaries. Pharmacoepidemiol Drug Saf.

[14] Nadkarni PM, Ohno-Machado L, Chapman WW (2011). Natural language processing: an introduction. J Am Med Inform Assoc.

[15] Yim WW, Yetisgen M, Harris WP, Kwan SW (2016). Natural language processing in oncology: a review. JAMA Oncol.

[16] Mehta N, Pandit A (2018). Concurrence of big data analytics and healthcare: A systematic review. Int J Med Inform.

[17] Li R, Hu B, Liu F (2019). Detection of bleeding events in electronic health record notes using convolutional neural network models enhanced with recurrent neural network autoencoders: deep learning approach. JMIR Med Inform.

[18] Tang B, Cao H, Wu Y, Jiang M, Xu H (2012). Clinical entity recognition using structural support vector machines with rich features. https://dl.acm.org/doi/proceedings/10.1145/2390068.

[19] Hao T, Huang Z, Liang L, Weng H, Tang B (2021). Health natural language processing: methodology development and applications. JMIR Med Inform.

[20] Hossain E, Rana R, Higgins N (2023). Natural language processing in electronic health records in relation to healthcare decision-making: a systematic review. Comput Biol Med.

[21] Locke S, Bashall A, Al-Adely S, Moore J, Wilson A, Kitchen GB (2021). Natural language processing in medicine: a review. Trends in Anaesthesia and Critical Care.

[22] Grootendorst M (2022). BERTopic: neural topic modeling with a class-based TF-IDF procedure. arXiv.

[23] McInnes L, Healy J, Melville J (2018). Umap: uniform manifold approximation and projection for dimension reduction. arXiv.

[24] Birant D, Kut A (2007). ST-DBSCAN: An algorithm for clustering spatial–temporal data. Data Knowl Eng.

[25] Schulman S, Kearon C, Subcommittee on Control of Anticoagulation of the Scientific and Standardization Committee of the International Society on Thrombosis and Haemostasis (2005). Definition of major bleeding in clinical investigations of antihemostatic medicinal products in non-surgical patients. J Thromb Haemost.

[26] Honnibal M, Montani I (2017). spaCy 2: Natural language understanding with Bloom embeddings, convolutional neural networks and incremental parsing. Sentometrics Research (forthcoming).

[27] Kiss T, Strunk J (2006). Unsupervised multilingual sentence boundary setection. Computational Linguistics.

[28] He J, Cheng MX (2021). Weighting methods for rare event identification from imbalanced datasets. Front Big Data.

[29] Moss HB, Leslie DS, Rayson P (2018). Using JK fold cross validation to reduce variance when tuning NLP models. arXiv.

[30] Liu Y, Lian J, Bartolacci MR, Zeng QA (2014). Density‐based penalty parameter optimization on C‐SVM. ScientificWorldJournal.

[31] Radzi SFM, Karim MKA, Saripan MI, Rahman MAA, Isa INC, Ibahim MJ (2021). Hyperparameter tuning and pipeline optimization via grid search method and tree-based AutoML in breast cancer prediction. J Pers Med.

[32] Walther D, Halfon P, Tanzer R (2021). Hospital discharge data is not accurate enough to monitor the incidence of postpartum hemorrhage. PLoS One.

[33] Hartenstein A, Abdelgawwad K, Kleinjung F, Privitera S, Viethen T, Vaitsiakhovich T (2023). Identification of International Society on Thrombosis and Haemostasis major and clinically relevant non-major bleed events from electronic health records: a novel algorithm to enhance data utilisation from real-world sources. Int J Popul Data Sci.

[34] Joos C, Lawrence K, Jones AE, Johnson SA, Witt DM (2019). Accuracy of ICD-10 codes for identifying hospitalizations for acute anticoagulation therapy-related bleeding events. Thromb Res.

[35] Metz CE (1978). Basic principles of ROC analysis. Semin Nucl Med.

[36] Sim J, Wright CC (2005). The kappa statistic in reliability studies: use, interpretation, and sample size requirements. Phys Ther.

[37] Sun W, Cai Z, Li Y, Liu F, Fang S, Wang G (2018). Data processing and text mining technologies on electronic medical records: a review. J Healthc Eng.

[38] Mitra A, Rawat BPS, McManus D, Kapoor A, Yu H (2020). Bleeding entity recognition in electronic health records: a comprehensive analysis of end-to-end systems. AMIA Annu Symp Proc.

[39] Alsentzer E, Murphy JR, Boag W (2019). Publicly available clinical BERT embeddings. arXiv.

[40] Li Y, Dong W, Ru B, Black A, Zhang X, Guan Y (2022). Generic medical concept embedding and time decay for diverse patient outcome prediction tasks. iScience.

[41] Lewis P, Ott M, Du J, Stoyanov V Pretrained language models for biomedical and clinical tasks: understanding and extending the state-of-the-art.

[42] Wu S, Roberts K, Datta S (2020). Deep learning in clinical natural language processing: a methodical review. J Am Med Inform Assoc.

[43] Zhou L, Hripcsak G (2007). Temporal reasoning with medical data--a review with emphasis on medical natural language processing. J Biomed Inform.

[44] Zhou L, Melton GB, Parsons S, Hripcsak G (2006). A temporal constraint structure for extracting temporal information from clinical narrative. J Biomed Inform.

[45] Jagannatha A, Liu F, Liu W, Yu H (2019). Overview of the first natural language processing challenge for extracting medication, indication, and adverse drug events from electronic health record notes (MADE 1.0). Drug Saf.

[46] Johnson SA, Signor EA, Lappe KL (2021). A comparison of natural language processing to ICD-10 codes for identification and characterization of pulmonary embolism. Thromb Res.

[47] Pedersen JS, Laursen MS, Rajeeth Savarimuthu T (2021). Deep learning detects and visualizes bleeding events in electronic health records. Res Pract Thromb Haemost.

[48] Taggart M, Chapman WW, Steinberg BA (2018). Comparison of 2 natural language processing methods for identification of bleeding among critically ill patients. JAMA Netw Open.

[49] Zeng Z, Deng Y, Li X, Naumann T, Luo Y (2019). Natural language processing for EHR-based computational phenotyping. IEEE/ACM Trans Comput Biol Bioinform.

[50] Dave T, Athaluri SA, Singh S (2023). ChatGPT in medicine: an overview of its applications, advantages, limitations, future prospects, and ethical considerations. Front Artif Intell.

[51] Ray S (2023). Segmented harmonic loss: handling class-imbalanced multi-label clinical data for medical coding with large language models. arXiv.

[52] Henning S, Beluch W, Fraser A, Friedrich A (2022). A survey of methods for addressing class imbalance in deep-learning based natural language processing. https://aclanthology.org/2023.eacl-main.

[53] Blanchard AE, Gao S, Yoon HJ (2022). A keyword-enhanced approach to handle class imbalance in clinical text classification. IEEE J Biomed Health Inform.

[54] Deng J, Zubair A, Park YJ (2023). Limitations of large language models in medical applications. Postgrad Med J.

[55] Sallam M, Salim NA, Barakat M, Al-Tammemi AB (2023). ChatGPT applications in medical, dental, pharmacy, and public health education: A descriptive study highlighting the advantages and limitations. Narra J.

[56] Chen Z, Cano AH, Romanou A (2023). MEDITRON-70B: Scaling medical pretraining for large language models. arXiv.

[57] Yang X, PourNejatian N, Shin HC (2022). GatorTron: a large language model for clinical natural language processing. medRxiv.

[58] Nori H, King N, McKinney SM (2023). Capabilities of GPT-4 on medical challenge problems. arXiv.

[59] Haupt CE, Marks M (2023). AI-Generated medical advice-GPT and beyond. JAMA.

[60] Head CB, Jasper P, McConnachie M, Raftree L, Higdon G (2023). Large language model applications for evaluation: Opportunities and ethical implications. New Drctns Evaluation.

