# Supplementary material for: Natural Language Processing and ICD-10 Coding for Detecting Bleeding Events in Discharge Summaries: Comparative Cross-Sectional Study
Source: JMIR Med Inform. 2025 Aug 29;13:e67837. doi: 10.2196/67837 (PMC12396801; doi:10.2196/67837)
Supplement: Multimedia Appendix 1 [file medinform-v13-e67837-s001.docx]

**Supplementary materials. A comparative cross-sectional study of Natural Language Processing and ICD-10 Coding for detecting bleeding events in discharge summaries:**

Frédéric Gaspar^1 2 3^, Mehdi Zayene^4^, Claire Coumau^1 2 3^, Zachary Schillaci^4^, Marie Bettex^5^, Elliott Bertrand^4^, Marie-Annick Le Pogam*^5^, Chantal Csajka*^#1 2 3^ and the SwissMADE study group.

^1^Center for Research and Innovation in Clinical Pharmaceutical Sciences, Lausanne University Hospital and University of Lausanne, Lausanne, Switzerland.

^2^School of Pharmaceutical Sciences, University of Geneva, Geneva, Switzerland.

^3^Institute of Pharmaceutical Sciences of Western Switzerland, University of Geneva, University of Lausanne, Geneva and Lausanne, Switzerland.

^4^Effixis SA, Lausanne, Switzerland

^5^Center for Primary Care and Public Health (Unisanté), Department of Epidemiology and Health Systems, University of Lausanne, Lausanne, Switzerland.

**Methods used to identify and select relevant discharge summaries**


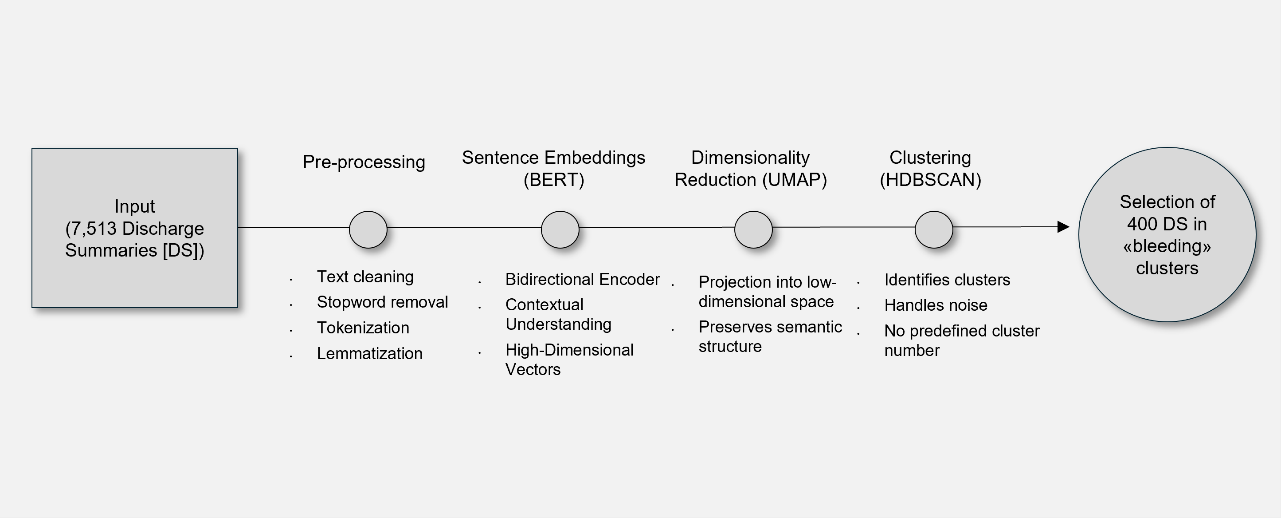


**Figure S1**: Methods for Identifying and Selecting Discharge Summaries

This appendix outlines the methodologies used to identify and select discharge summaries of interest. The dataset of 7,513 summaries was processed using advanced natural language processing (NLP) and machine learning techniques to detect potential bleeding Adverse Drug Events (ADEs).

The first step involved pre-processing the discharge summaries to prepare them for analysis. This included text cleaning, where irrelevant sections such as headers, footers, and administrative information were removed. Additionally, stopwords, punctuation, and other non-informative elements were eliminated to streamline the dataset. Tokenization was applied to split the text into individual tokens, which served as the basic units for subsequent NLP analysis. Lemmatization followed, reducing words to their base or root form to ensure consistency in the data representation. This step was critical to treating variations of the same word (e.g., “bleeding” vs. “bleed”) as equivalent during the analysis.

Once the text was pre-processed, sentence embeddings were generated to capture the semantic meaning of the text. Bidirectional Encoder Representations from Transformers (BERT) were utilized to create these embeddings. BERT is a state-of-the-art NLP model capable of understanding context bidirectionally, making it particularly suitable for complex clinical text. The embeddings generated by BERT transformed each sentence into a high-dimensional vector, effectively capturing the nuances in medical language, such as distinguishing between “clinically significant bleeding” and “no evidence of bleeding” [1]. This representation allowed the model to recognize subtle differences in clinical phrases that could be overlooked by traditional keyword-based methods.

To manage the high dimensionality of the embeddings and facilitate clustering, Uniform Manifold Approximation and Projection (UMAP) was applied for dimensionality reduction. UMAP is a non-linear reduction technique designed to preserve both local and global structure within the data. It projects the high-dimensional embeddings into a lower-dimensional space, typically two or three dimensions, while maintaining the relative distances between sentences based on their semantic similarities. This step was crucial in organizing the data for clustering and for better visualizing the relationships between different discharge summaries [2].

The reduced-dimensional data were then subjected to a clustering algorithm. Hierarchical Density-Based Spatial Clustering of Applications with Noise (HDBSCAN) was employed to group sentences and discharge summaries based on their proximity in the reduced space. HDBSCAN is particularly advantageous in medical data analysis due to its ability to identify clusters of varying shapes and densities without requiring a predefined number of clusters. Furthermore, it can effectively manage noise and outliers, ensuring that only clinically meaningful groups of discharge summaries are retained for further evaluation [3]. This was essential given the complexity and variability of clinical narratives.

Following the clustering process, 400 discharge summaries were identified as likely to contain bleeding ADEs (within bleeding clusters). These summaries were randomly selected to ensure a representative sample of the overall dataset. The selected summaries were then subjected to a detailed clinical review by a panel of experienced clinicians, who manually verified the presence of bleeding ADEs.

**References**

1. Devlin, J., Chang, M.W., Lee, K., & Toutanova, K. (2018). BERT: Pre-training of deep bidirectional transformers for language understanding. *arXiv preprint arXiv:1810.04805*.
2. McInnes, L., Healy, J., & Melville, J. (2018). UMAP: Uniform manifold approximation and projection for dimension reduction. *arXiv preprint arXiv:1802.03426*.
3. Campello, R.J.G.B., Moulavi, D., & Sander, J. (2013). Density-based clustering based on hierarchical density estimates. *Advances in Knowledge Discovery and Data Mining*, 160-172.

**Addressing Class Imbalance in the dataset**

This appendix details the strategies implemented to manage class imbalance in the dataset, a critical consideration when detecting rare but clinically significant events such as bleeding. Class imbalance is a common challenge in medical datasets, where majority classes often dominate, overshadowing minority classes that represent important clinical conditions.

Figure S2 illustrates the class distributions at both the document and sentence levels, highlighting the imbalances observed in this study and their implications for the classification process.


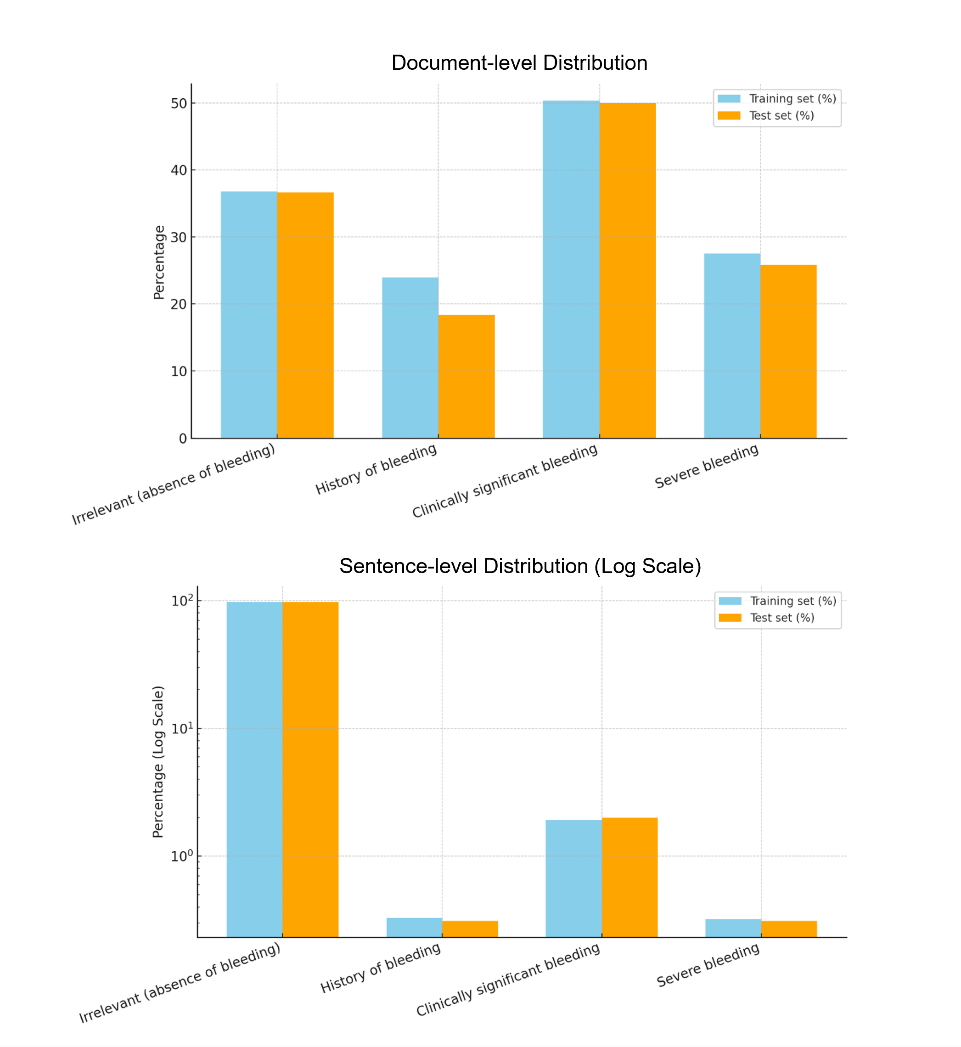


**Figure S2:** Distribution of bleeding labels in training and test sets at document and sentence levels

At the document level, the dataset was balanced across bleeding classifications through an unsupervised selection approach, as detailed in the previous section. This balanced representation enabled the machine learning models to generalize effectively during document-level classification tasks. In contrast, at the sentence level, the dataset exhibited significant imbalance, with the "irrelevant" class dominating the labels. The "irrelevant" class comprised sentences that lacked bleeding-related information, constituting the majority of the dataset. This posed challenges during the first-stage binary classification, where distinguishing "irrelevant" from "relevant" was critical for filtering non-bleeding information. To address this imbalance during the training of the first-stage binary classifier ("irrelevant" vs. "relevant"), class weighting was applied in the logistic regression model [1,2]. This technique adjusts the contribution of each class to the loss function by assigning weights inversely proportional to their frequencies in the training data. Specifically, for a dataset with *n* total samples and *n_c_​* samples in
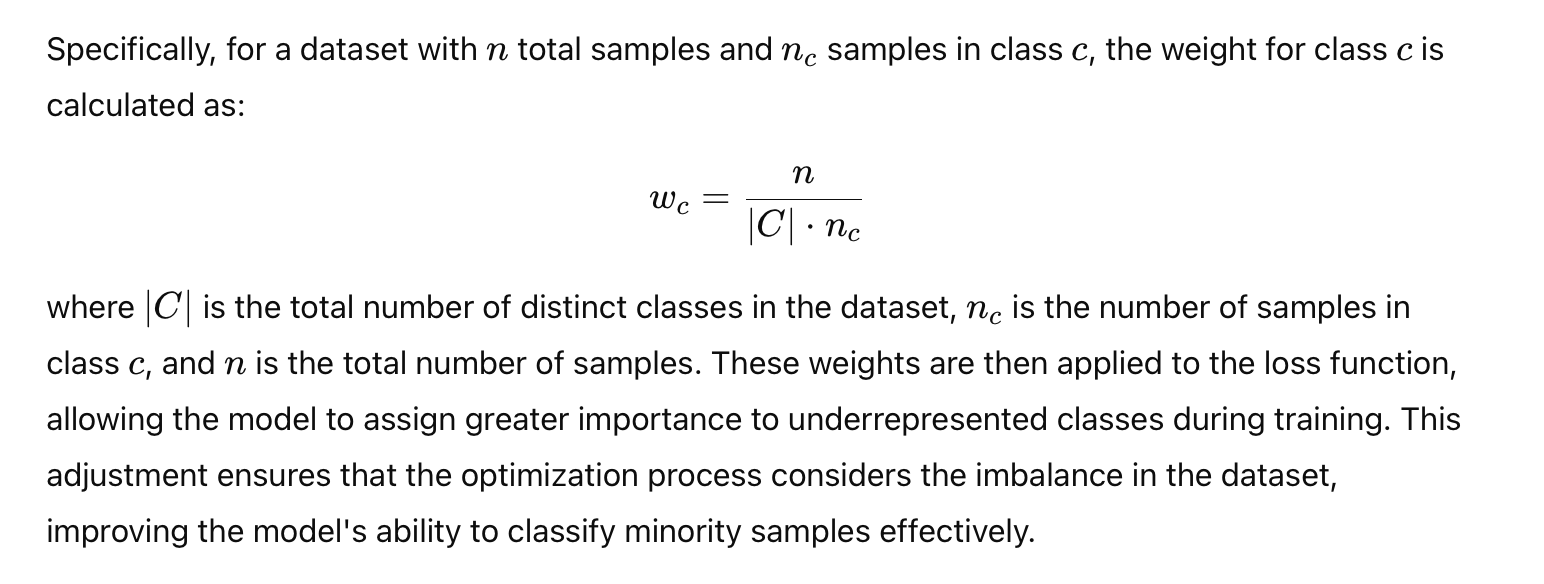
class ∣C∣, the weight for class *w_c_* is calculated as:

where ∣C∣ is the total number of distinct classes in the dataset, *n_c_​* ​ is the number of samples in class *c*, and *n* is the total number of samples. These weights were applied to the loss function to give greater importance to underrepresented classes, effectively improving the model's ability to classify minority samples. This adjustment ensured that the optimization process considered the inherent imbalance, allowing for better detection of rare but clinically critical cases, such as severe bleeding [3].

**References**

1. Chawla, N. V., Bowyer, K. W., Hall, L. O., & Kegelmeyer, W. P. (2002). SMOTE: Synthetic Minority Over-sampling Technique. Journal of Artificial Intelligence Research, 16, 321-357. DOI: 10.1613/jair.953
2. He, H., & Garcia, E. A. (2009). Learning from Imbalanced Data. IEEE Transactions on Knowledge and Data Engineering, 21(9), 1263-1284. DOI: 10.1109/TKDE.2008.239
3. Buda, M., Maki, A., & Mazurowski, M. A. (2018). A systematic study of the class imbalance problem in convolutional neural networks. Neural Networks, 106, 249-259. DOI: 10.1016/j.neunet.2018.07.011

**Table S1**: ICD-10 Codes related to severe bleeding

| ICD10GM Code | ICD10GM 2 Code 2 | Root Code | Description - Severe bleeding | Location1 | Acronym1 | Location2 | Acronym2 | Critical Organ or Area |
| --- | --- | --- | --- | --- | --- | --- | --- | --- |
| H21.0 | H210 | 0 | Hyphema | Intraocular | INTO | Other critical site | OCS | 1 |
| H31.3 | H313 | 0 | Choroid haemorrhage and rupture | Intraocular | INTO | Other critical site | OCS | 1 |
| H35.6 | H356 | 0 | Retinal haemorrhage | Intraocular | INTO | Other critical site | OCS | 1 |
| H43.1 | H431 | 0 | Vitreous haemorrhage | Intraocular | INTO | Other critical site | OCS | 1 |
| H45.0 | H450 | 0 | Vitreous haemorrhage in diseases classified elsewhere | Intraocular | INTO | Other critical site | OCS | 1 |
| I23.0 | I230 | 0 | Haemopericardium as a recent complication of acute myocardial infarction | Cardial | CARD | Other critical site | OCS | 1 |
| I31.2 | I312 | 0 | Haemopericardium not elsewhere classified | Cardial | CARD | Other critical site | OCS | 1 |
| I60_ | I60 | 1 | Subarachnoid haemorrhage | Intracranial | CNS | Intracranial | CNS | 1 |
| I61_ | I61 | 1 | Intracerebral haemorrhage | Intracranial | CNS | Intracranial | CNS | 1 |
| I62_ | I62 | 1 | Other nontraumatic intracranial haemorrhages | Intracranial | CNS | Intracranial | CNS | 1 |
| I67.80 | I6780 | 0 | Vasospasm during subarachnoid haemorrhage | Intracranial | CNS | Intracranial | CNS | 1 |
| K66.1 | K661 | 0 | Haemoperitoneum | Peritoneal | RPRT | Other critical site | OCS | 1 |
| M25.0_ | M250 | 1 | Haemarthrosis | Intraarticular | INTART | Other critical site | OCS | 1 |
| S06.23 | S0623 | 0 | Multiple cerebral and cerebellar haematomas | Intracranial | CNS | Trauma | TR | 1 |
| S06.33 | S0633 | 0 | Localised cerebral haematoma | Intracranial | CNS | Trauma | TR | 1 |
| S06.34 | S0634 | 0 | Localised cerebellar haematoma | Intracranial | CNS | Trauma | TR | 1 |
| S06.4 | S064 | 0 | Epidural haemorrhage | Intracranial | CNS | Trauma | TR | 1 |
| S06.5 | S065 | 0 | Traumatic subdural haemorrhage | Intracranial | CNS | Trauma | TR | 1 |
| S06.6 | S066 | 0 | Traumatic subarachnoid haemorrhage | Intracranial | CNS | Trauma | TR | 1 |
| S06.8 | S068 | 0 | Other traumatic intracranial injuries | Intracranial | CNS | Trauma | TR | 1 |

This table presents the ICD-10 codes associated with severe bleeding. It includes detailed descriptions of the bleedings, the anatomical locations affected, as well as the critical organs or systems involved.

**Table S2**: ICD-10 Codes related to clinically significant bleeding

| ICD10GM Code | ICD10GM 2 Code 2 | Root Code | Description - clinically significant bleeding | Location1 | Acronym1 | Location2 | Acronym2 | Critical Organ or Area |
| --- | --- | --- | --- | --- | --- | --- | --- | --- |
| K57.11 | K5711 | 0 | Diverticulosis of small intestine without perforation or abscess, with bleeding | Gastrointestinal | GI | Gastrointestinal | GI | 0 |
| K57.13 | K5713 | 0 | Diverticulitis of small intestine without perforation or abscess, with bleeding | Gastrointestinal | GI | Gastrointestinal | GI | 0 |
| K57.31 | K5731 | 0 | Diverticulosis of colon without perforation or abscess, with bleeding | Gastrointestinal | GI | Gastrointestinal | GI | 0 |
| K57.33 | K5733 | 0 | Diverticulitis of colon without perforation or abscess, with bleeding | Gastrointestinal | GI | Gastrointestinal | GI | 0 |
| K62.5 | K625 | 0 | Haemorrhage of anus and rectum | Gastrointestinal | GI | Gastrointestinal | GI | 0 |
| K92.0 | K920 | 0 | Haematemesis | Gastrointestinal | GI | Gastrointestinal | GI | 0 |
| K92.1 | K921 | 0 | Melaena | Gastrointestinal | GI | Gastrointestinal | GI | 0 |
| K92.2 | K922 | 0 | Gastrointestinal haemorrhage, unspecified | Gastrointestinal | GI | Gastrointestinal | GI | 0 |
| N02._ | N02 | 1 | Recurrent and persistent haematuria | Urogenital | UROGEN | Other non-critical site | ONCS | 0 |
| N42.1 | N421 | 0 | Prostatic congestion and haemorrhage | Urogenital | UROGEN | Other non-critical site | ONCS | 0 |
| N83.6 | N836 | 0 | Haematosalpinx | Urogenital | UROGEN | Other non-critical site | ONCS | 0 |
| N83.7 | N837 | 0 | Haematoma of broad ligament | Urogenital | UROGEN | Other non-critical site | ONCS | 0 |
| N85.7 | N857 | 0 | Haematometra | Urogenital | UROGEN | Other non-critical site | ONCS | 0 |
| N89.7 | N897 | 0 | Haematocolpos | Urogenital | UROGEN | Other non-critical site | ONCS | 0 |
| N92.0 | N920 | 0 | Excessive and frequent menstruation with regular cycle | Urogenital | UROGEN | Other non-critical site | ONCS | 0 |
| N92.1 | N921 | 0 | Excessive and frequent menstruation with irregular cycle | Urogenital | UROGEN | Other non-critical site | ONCS | 0 |
| N92.4 | N924 | 0 | Pre-menopausal excessive bleeding | Urogenital | UROGEN | Other non-critical site | ONCS | 0 |
| N93._ | N93 | 1 | Other specified abnormal uterine and vaginal bleeding | Urogenital | UROGEN | Other non-critical site | ONCS | 0 |
| N95.0 | N950 | 0 | Postmenopausal bleeding | Urogenital | UROGEN | Other non-critical site | ONCS | 0 |
| R04.0 | R040 | 0 | Epistaxis | Ear-Nose-Throat | ENT | Other non-critical site | ONCS | 0 |
| R04.1 | R041 | 0 | Throat haemorrhage | Ear-Nose-Throat | ENT | Other non-critical site | ONCS | 0 |
| R04.2 | R042 | 0 | Haemoptysis | Respiratory tract | RESP | Other non-critical site | ONCS | 0 |
| R04.8 | R048 | 0 | Haemorrhage from other parts of respiratory tract | Respiratory tract | RESP | Other non-critical site | ONCS | 0 |
| R04.9 | R049 | 0 | Haemorrhage of respiratory tract, unspecified | Respiratory tract | RESP | Other non-critical site | ONCS | 0 |
| R23.3 | R233 | 0 | Spontaneous bruising | Cutaneous | CUT | Other non-critical site | ONCS | 0 |
| R31 | R31 | 0 | Haematuria, unspecified | Urogenital | UROGEN | Other non-critical site | ONCS | 0 |
| R58 | R58 | 0 | Haemorrhage, not elsewhere classified | Unspecified site | UNSP | Unspecified site | UNSP | NA |
| T81.0 | T810 | 0 | Haemorrhage and haematoma complicating a diagnostic or therapeutic procedure | Unspecified site | UNSP | Unspecified site | UNSP | NA |
| T79.2 | T792 | 0 | Secondary and recurrent traumatic haemorrhage | Unspecified site | UNSP | Unspecified site | UNSP | NA |

This table presents the ICD-10 codes associated with clinically significant bleeding. It includes detailed descriptions of the bleedings, the anatomical locations affected, as well as the critical organs or systems involved.
